# Comparative In Vitro Drug Susceptibility Study of Five Oxazolidinones Against *Mycobacterium tuberculosis* in Hainan, China

**DOI:** 10.3390/pathogens14030218

**Published:** 2025-02-24

**Authors:** Jinhui Dong, Qian Cheng, Chuanning Tang, Yeteng Zhong, Jieying Wang, Meiping Lv, Zhuolin Chen, Peibo Li, Ming Luo, Hua Pei

**Affiliations:** 1 Department of Clinical Laboratory, The Second Affiliated Hospital of Hainan Medical University, Haikou 571199, China; Dong18515940918@126.com (J.D.); zhongyeteng@126.com (Y.Z.); wangjieying765@126.com (J.W.); lmp_hnykdx@126.com (M.L.); czl305818060@126.com (Z.C.); 2 Tuberculosis Research Units, Chongqing Public Health Medical Center, Chongqing 400036, China; chengqianc2024@126.com (Q.C.); lpb2025@126.com (P.L.); 3 Hainan Medical University-The University of Hong Kong Joint Laboratory of Tropical Infectious Diseases, Key Laboratory of Tropical Translational Medicine of Ministry of Education, Hainan Medical University, Haikou 571199, China; chuanningtang@hotmail.com; 4 Clinical Laboratory, Chongqing Public Health Medical Center, Chongqing 400036, China

**Keywords:** oxazolidinones, *Mycobacterium tuberculosis*, minimum inhibitory concentration, drug resistance

## Abstract

Oxazolidinones, novel synthetic antibacterials, inhibit protein biosynthesis and show potent activity against Gram-positive bacteria, including *Mycobacterium tuberculosis* (MTB). In this study, we aimed to compare the in vitro activity of linezolid (LZD) and four oxazolidinones, including tedizolid (TZD), contezolid (CZD), sutezolid (SZD), and delpazolid (DZD), against multidrug-resistant tuberculosis (MDR-TB) and pre-extensively drug-resistant tuberculosis (pre-XDR-TB) isolates from Hainan. We established their epidemiological cut-off values (ECOFFs) using ECOFFinder software and analyzed mutations in *rrl* (23S rRNA), *rplC*, *rplD*, *mce3R*, *tsnR*, *Rv0545c*, *Rv0930*, *Rv3331*, and *Rv0890c* genes to uncover potential mechanisms of oxazolidinone resistance. This study included 177 MTB isolates, comprising 67 MDR and 110 pre-XDR-TB isolates. Overall, SZD exhibited the strongest antibacterial activity against clinical MTB isolates, followed by TZD and LZD, with CZD and DZD showing equivalent but weaker activity (SZD_MIC50_ = TZD_MIC50_ < LZD_MIC50_ < CZD_MIC50_ = DZD_MIC50_; SZD_MIC90_ < TZD_MIC90_ = LZD_MIC90_ < CZD_MIC90_ = DZD_MIC90_). Significant differences in MIC distribution were observed for TZD (*p* < 0.0001), CZD (*p* < 0.01), SZD (*p* < 0.0001), and DZD (*p* < 0.0001) compared to LZD but not between MDR-TB and pre-XDR-TB isolates. We propose the following ECOFFs: SZD, 0.5 µg/mL; LZD, TZD, and CZD, 1.0 µg/mL; DZD, 2.0 µg/mL. No statistically significant differences in resistance rates were observed among these five drugs (*p* > 0.05). We found that eight MTB isolates (4.52% [8/177]) resisted these five oxazolidinones. Among these, only one isolate, M26, showed an amino acid substitution (Arg79His) in the protein encoded by the *rplD* gene, which conferred cross-resistance to TZD and CZD. Three distinct mutations were identified in the *mce3R* gene; notably, isolate P604 displayed two insertions that contributed to resistance against all five oxazolidinones. However, no significant correlation was observed between mutations in the *rrl*, *rplC*, *rplD*, *mce3R*, *tsnR*, *Rv0545c*, *Rv0930*, *Rv3331*, and *Rv0890c* genes with oxazolidinone resistance in the clinical MTB isolates tested. In summary, this study provides the first report on the resistance of MTB in Hainan to the five oxazolidinones (LZD, TZD, CZD, SZD, and DZD). In vitro susceptibility testing indicated that SZD exhibited the strongest antibacterial activity, followed by TZD and LZD, while CZD and DZD demonstrated comparable but weaker effectiveness. Mutations in *rplD* and *mce3R* were discovered, but further research is needed to clarify their role in conferring oxazolidinone resistance in MTB.

## 1. Introduction

Tuberculosis (TB), an infectious disease caused by *Mycobacterium tuberculosis* (MTB), remains the leading single cause of death worldwide [1]. An estimated 10.8 million new cases of TB and 1.25 million deaths from TB will occur worldwide in 2023, including 400,000 new cases of multidrug- and rifampicin-resistant TB, with about 29,000 cases in our country (7.3%) [1]. Compared with drug-sensitive TB, treatment regimens for drug-resistant TB, particularly multidrug-resistant TB (MDR-TB), are characterized by longer disease duration, greater economic burden, and increased drug toxicity, resulting in severe adverse effects and poorer treatment outcomes [2]. Therefore, MDR-TB remains a major public health problem for the treatment and control of TB, and there is an urgent need for repurposed anti-TB drugs and new drugs to treat drug-resistant TB more safely and effectively.

**Figure 1:**
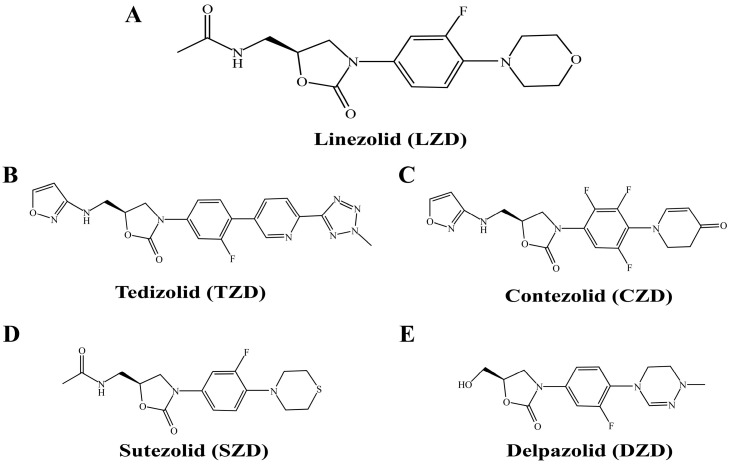
Chemical structures of five oxazolidinones. (**A**): Linezolid (LZD); (**B**): Tedizolid (TDZ); (**C**): Contezolid (CZD); (**D**): Sutezolid (SZD); (**E**): Delpazolid (DZD).

Oxazolidinones are a class of synthetic antibiotics active against Gram-positive bacteria. Linezolid (LZD) is the first oxazolidinone antibiotic to be used in clinical practice (Figure 1A); its mechanism of action is to inhibit bacterial protein synthesis at an early stage of translation by binding to the 23S ribosomal mRNA of the 50S subunit of the mycobacterial ribosome, providing an anti-tuberculosis effect [3]. LZD has shown effective activity against drug-resistant MTB in both in vitro and in vivo studies, and in 2020, the WHO included LZD in the standard long-term and short-term treatment regimens for M/XDR-TB [4]. However, clinical studies have shown a high incidence of adverse events (AEs) in MDR-TB patients treated with LZD [4,5], with common adverse effects including peripheral neuropathy, bone marrow suppression, and varying degrees of anemia. Due to the significant adverse effects of LZD, there is an urgent need to explore other compounds within the oxazolidinone class of drugs to develop safer and more effective alternatives for drug-resistant TB treatment.

Tedizolid (TDZ), Contezolid (CZD), Sutezolid (SZD), and Delpazolid (DZD) represent the latest oxazolidinone drugs in development, showing considerable potential for combating drug-resistant tuberculosis (Figure 1B–E). TDZ, also known as TR-700 or DA-7157, belongs to the second generation of oxazolidinones and distinguishes itself with a unique D-ring structure and a hydroxymethyl C-5 side chain when compared to LZD, resulting in enhanced efficacy against LZD-resistant pathogens [6,7,8]. Tedizolid phosphate, developed by Cubist Pharmaceuticals, received approval in the United States in 2014 for treating acute bacterial and skin structure infections [9]. CZD was specifically formulated to address MDR Gram-positive bacterial infections, notably methicillin-resistant Staphylococcus aureus (MRSA), and was authorized for use in China in 2021 for the management of complex skin and soft tissue infections [10]. SZD, formerly recognized as PNU-100480, represents a thiomorpholine oxazolidinone and a structural analog of LZD. It distinguishes itself from LZD by incorporating a sulfur atom instead of an oxygen atom within its ring structure [11]. DZD (LCB01-0371) emerges as a novel oxazolidinone created by LegoChem BioSciences Inc. in Daejeon, Republic of Korea, featuring a cyclic amidrazone that facilitates gradual drug accumulation and efficient excretion, potentially minimizing long-term adverse effects [12].

Oxazolidinones play a crucial role in combating drug-resistant tuberculosis, with current knowledge of resistance mechanisms primarily focused on LZD. The prevalent mechanism of LZD resistance involves mutations in ribosomal components near the peptidyltransferase center (PTC) [13,14], commonly found in the *rplC* gene, *rplD* gene [15], 23S sRNA (*rrl*) [16], and genes encoding ribosomal enzymes such as *tsnR* [17]. Recent research has shed light on non-ribosomal resistance mechanisms in LZD involving efflux pump/transporter proteins, such as *Rv0545c*, *Rv0930*, *Rv3331*, and transcriptional regulator *Rv0890c* [18]. In addition, in the study of CZD resistance mechanisms, mutations in *mce3R* were found to upregulate the expression of a possible xanthine monooxygenase-encoding gene, *Rv1936*, resulting in MTB resistance to CZD [19]. However, data on the efficacy of the oxazolidinones against MDR and pre-XDR-TB strains and potential resistance mechanisms are still limited. In this study, we compared the in vitro activity of the four oxazolidinones (TZD, CZD, SZD, and DZD) with LZD against MDR and pre-XDR-TB isolates in Hainan. Additionally, an analysis of mutations in *rrl*, *rplC*, *rplD*, *mce3R*, *tsnR*, *Rv0545c*, *Rv0930*, *Rv3331*, and *Rv0890c* was conducted to investigate potential oxazolidinone resistance mechanisms in MTB.

## 2. Materials and Methods

### 2.1. Ethics Statement

Because this study involved only laboratory testing with reference strains and clinical isolates, no ethical approval was sought. This study was conducted as a secondary analysis, and since the data did not contain any identifying information about individuals, the requirement for informed consent was waived.

### 2.2. Clinical Isolates and Culture Conditions

A total of 177 selected multidrug-resistant clinical isolates were obtained from patients consecutively admitted to a medical service in the Second Affiliated Hospital of Hainan Medical University (Hainan, China) between July 2019 and July 2022. The comprehensive information was gathered and documented for all patients, including age, gender, TB contact history, and TB treatment history. Each MTB isolate was isolated from a unique patient. Drug susceptibility was determined using the Roche proportion method on Löwenstein–Jensen (L-J) medium containing the corresponding anti-TB drugs according to the guidelines of the WHO recommendations [20]. Concentrations of anti-TB drugs in L-J medium were as follows: isoniazid (INH, 0.2 µg/mL), rifampin (RIF, 40.0 µg/mL), ethambutol (EMB, 2.0 µg/mL), capreomycin (CPM, 2.0 µg/mL), kanamycin (KM, 40.0 µg/mL), ofloxacin (30.0 µg/mL), and protionamide (PTO, 40.0 µg/mL). MDR-TB was defined as TB that is resistant to INH and RIF. Pre-XDR-TB was defined as MDR-TB with additional resistance to any fluoroquinolone [21]. All bacterial cells were stored in 7H9 broth containing 15% glycerol in a −80 °C refrigerator. Before in vitro susceptibility testing, the isolates were recovered on L-J medium for 4 weeks at 37 °C.

### 2.3. Minimum Inhibitory Concentration Testing (MIC) Determinations

The microplate Alamar Blue assay (MABA) was performed to determine the MICs of MDR-TB isolates against oxazolidinones [22]. Briefly, 100 µL of 7H9 broth containing varying concentrations of oxazolidinones (LZD, TZD, CZD, SZD, or DZD) were added to the sample wells of a 96-well plate. These drugs were serially diluted, with the last well serving as a drug-free blank control. The final concentration of the five drugs ranged from 0.032 to 32 µg/mL (0.032, 0.063, 0.125, 0.25, 0.5, 1, 2, 4, 8, 16, and 32 µg/mL). Bacterial clones were harvested from the surfaces of L-J slants. After vigorous mixing on a vortex mixer for 1 min, the suspension was adjusted to a turbidity equivalent to 1.0 McFarland. The inoculum was further prepared by 1:20 dilution of cell suspension with Middlebrook 7H9 broth containing 10% oleic acid-albumin-dextrose-catalase (OADC), and 100 μL of this inoculum was added to the wells of the 96-well plate containing the corresponding drugs. After 7 days of incubation, 70 μL of Alamar Blue solution (20 µL resazurin and 50 µL 5% Tween-80) was added to each well and incubated at 37 °C for 24 h. The color change was used to evaluate bacterial growth, and the MIC was defined as the lowest concentration of drug that prevented the color change from blue to pink. Each sample was tested in duplicate to ensure consistent results.

### 2.4. Determination of Epidemiological Cut-Off Values (ECOFF)

The ECOFF was determined by a combination of ’intuitive method’ and ’statistical method’. The visual estimation is to observe the histogram of the MIC distribution of clinical isolates. The ECOFF value is usually the MIC value of 1 to 2 dilutions after the peak value. Statistical method refers to the Clinical & Laboratory Standards Institute (CLSI) and recommended ECOFFinder software XL 2010 v2.1 (https://clsi.org/meetings/susceptibility-testing-subcommittees/ecoffinder/ (accessed on 24 December 2024)) was used for statistical analysis, which simulates ECOFF at 95%, 97.5%, 99%, 99.5%, and 99% confidence intervals. The ECOFF values of five oxazolidinone drugs (LZD, TZD, CZD, SZD, and DZD) were determined by combining these two methods.

### 2.5. Whole-Genome Sequencing

As previously reported, the fresh bacteria were harvested from the surface of an L-J slant and then transferred into a microcentrifuge tube containing 500 μL Tris-EDTA (TE) buffer for high-temperature inactivation at 80 °C for 30 min. DNA from in vitro evolution experiments was isolated using the cetyltrimethylammonium bromide method and sequenced on the Illumina NextSeq500 platform (Tibikang Biotechnology, Guangdong, China). DNA libraries were constructed and processed using Illumina kit according to the manufacturer’s instructions and aligned to the H37Rv reference genome (GenBank number NC_000962.3) using the SAM-TB platform. The sequences of genes known to confer oxazolidinone resistance in MTB (including *rrl*, *rplC*, *rplD*, *mce3R*, *tsnR*, *Rv0545c*, *Rv0930*, *Rv3331*, and *Rv0890c* genes) were specifically analyzed.

### 2.6. Statistical Analysis

The Pearson chi-square or Fisher’s exact test was used to compare proportions or rates. Each violin plot presents the distribution and probability density of the data. Statistical analysis was performed using SPSS version 26.0 software (SPSS Inc., Chicago, IL, USA). Differences were considered statistically significant for *p* < 0.05.

## 3. Results

### 3.1. Demographic and Clinical Characteristics of Study Participants

Among the 177 clinical isolates examined in this study, an assessment was conducted on the demographic characteristics of the patients, including gender, age, occupation, specimen type, and drug susceptibility, as shown in Table 1. The gender distribution showed 142 (80.2%, 95% CI, 75.4–87.3%) male patients and 35 (19.8%, 95% CI, 12.7–24.6%) female patients, with four times as many male patients as female patients. Analysis of the age distribution showed that all isolates originated from patients aged 15–80 years, with a mean age of 50 years. In addition, the majority of the TB patients enrolled in this study were local workers, with 26.6% classified as new cases, and sputum was the predominant specimen source. Drug susceptibility testing showed that ethambutol (EMB) had the highest resistance rate at 62.1%. The raw data are shown in Supplementary Table S1.

### 3.2. Oxazolidinone Activities Against Mycobacterium tuberculosis Isolates

**Figure 2:**
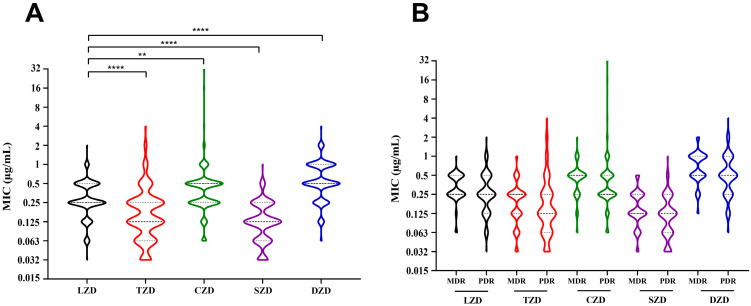
MIC distributions of oxazolidinones against *Mycobacterium tuberculosis*. In vitro, the antibacterial activity of oxazolidinones against MTB (**A**) and MDR-TB or pre-XDR-TB. (**B**) Date is presented by violin plot. Different colored plots represent different drugs. Nonparametric tests and Dunnett multiple comparison tests are used to compare statistical differences. ** *p* < 0.01, **** *p* < 0.0001. Abbreviations: MIC, minimum inhibitory concentration; MDR, multidrug-resistant TB; PDR. pre-extensively resistant TB.

The MICs of the five oxazolidinone drugs against 177 MTB isolates, including 67 MDR-TB isolates and 110 pre-XDR-TB isolates, are detailed in Table 2 and Figure 2. The susceptibility of MTB isolates to TZD was indicated by a MIC_50_ value of 0.125 µg/mL, which was 2-fold lower than the corresponding LZD value. Meanwhile, the median TZD MIC was 0.125 [0.063, 0.25] µg/mL, while the LZD MIC was 0.25 [0.125, 0.5] µg/mL (*p* < 0.0001). Susceptibility to SZD in MTB isolates was represented by the MIC_50_ and MIC_90_ values of 0.125 µg/mL and 0.25 µg/mL, respectively, which were 2-fold lower than the corresponding LZD values, and the median SZD MIC was 0.125 µg/mL and 2-fold lower than LZD (*p* < 0.0001). In addition, the susceptibility of MTB isolates to CZD and DZD was demonstrated by identical MIC_50_ and MIC_90_ values of 0.5 µg/mL and 1 µg/mL, respectively, which were twice as high as the corresponding LZD values. The median CZD and DZD MIC was 0.5 [0.25, 1] µg/mL, which was also twice as high as that of the LZD (CZD: *p* < 0.01, DZD: *p* < 0.0001). Furthermore, the MIC distributions of these five oxazolidinone drugs (LZD, TZD, CZD, SZD, and DZD) were not significantly different between MDR-TB and pre-XDR-TB (Figure 2B).

**Figure 3:**
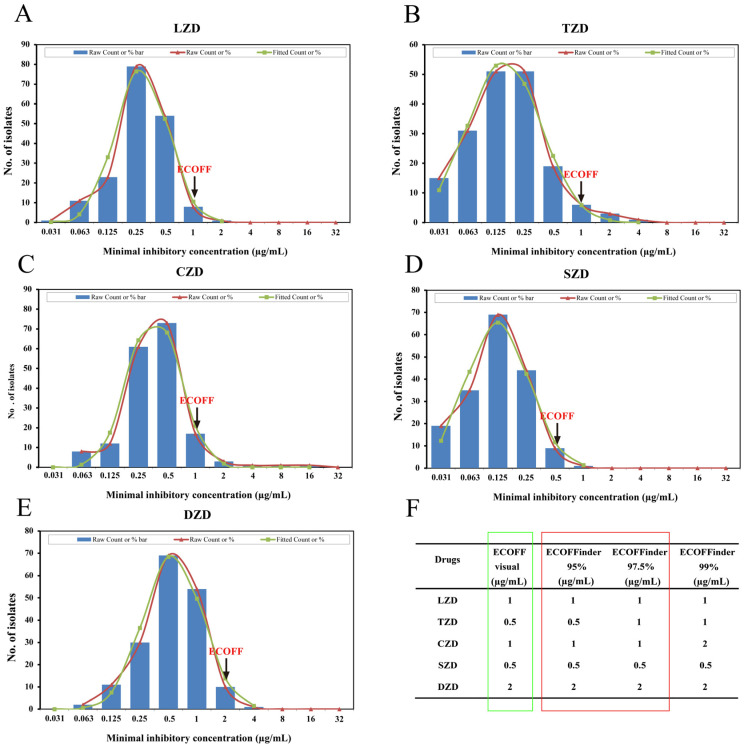
Non-linear regression analysis of the MIC distribution of five oxazolidinone drugs against MTB isolates: (**A**): Linezolid (LZD); (**B**): Tedizolid (TDZ); (**C**): Contezolid (CZD); (**D**): Sutezolid (SZD); (**E**): Delpazolid (DZD). The abscissa is the MIC value, and the ordinate is the number of isolates corresponding to each MIC. The histogram is the original MlC distribution peak, the red ‘Raw Count or %’ represents measured MIC data, and the green ‘Fitted Count or %’ represents simulated MIC data. The arrows on the plot highlight the proposed epidemiological cut-off values (ECOFF) for LZD, TZD, CZD, SZD, and DZD against MTB isolates. (**F**) The epidemiological cutoff values (ECOFF) for the five drugs (LZD, TZD, CZD, SZD, DZD) and the calculated values by ECOFFinder at different confidence levels (95%, 97.5%, 99%).

We further analyzed the provisional epidemiological cut-off values (ECOFF) for these five oxazolidinone drugs. The MIC values for these drugs were each found to have a unimodal distribution, as depicted in Figure 3. The ECOFF determination process involved using an intuitive method and calculating corresponding epidemiological critical values (ECVs) with different confidence intervals using the ECOFFinder software. Based on the results obtained through the intuitive method (Figure 3F), the ECOFF values at the 97% confidence interval were selected as the provisional ECOFF values for the MIC of the five oxazolidinone drugs. The results showed that the provisional ECOFFs for MIC were identified as 1.0 µg/mL for LZD, TZD, and CZD; 0.5 µg/mL for SZD; and 2.0 µg/mL for DZD (Figure 3A–E). Remarkably, the ECOFF for LZD corresponded to breakpoints used to define in vitro LZD resistance in previous studies.

Using a cut-off value of 1.0 µg/mL, resistance to LZD, TZD, and CZD was observed in one (0.56% [1/177 isolates]), four (2.26% [4/177 isolates]), and six (3.39% [6/177 isolates]) MTB isolates, respectively. Resistance to SZD was observed in one (0.56% [1/177 isolates]) MTB isolate at a cut-off value of 0.5 µg/mL. For DZD, resistance was observed in one (0.56% [1/177 isolates]) MTB isolate. A total of eight isolates (4.52% [8/177 isolates]) of MTB were resistant to these five oxazolidinones, with one isolate showing cross-resistance to the five oxazolidinones (LZD, TZD, CZD, SZD, and DZD) and one isolate showing cross-resistance to TZD and CZD. Of the single-resistant isolates, two were resistant to TZD, and four were resistant to CZD. Statistical analyses showed that the proportions of TZD-, CZD-, SZD-, and DZD-resistant isolates were not significantly different from those of LZD-resistant isolates (*p* > 0.5).

### 3.3. Mutations Conferring Resistance to Five Oxazolidinones

WGS was performed on 177 MTB isolates, including 8 oxazolidinone-resistant isolates, to identify potential mutations associated with oxazolidinone resistance. As shown in Table 3, the analysis revealed no nucleotide mutations in the *rrl* and *rplC* genes in these resistant isolates. For the *rplD* locus, a non-synonymous mutation, Arg79His (CGT→CAT), was identified on one isolate named M26, displaying resistance to both TZD and CZD. Moreover, three different mutation types were observed in the *mce3R* gene. Of these, two mutation types were located in the intergenic region of the P604-resistant isolate, consisting of a C-T mutation at position −723/−175 and a C insertion at positions −789/−109. Notably, the ins_C mutation at −789/−109 was also observed in the remaining six oxazolidinone-resistant isolates. Only the M26-resistant isolate harbored another non-synonymous mutation, Tyr73Asp (TAC→GAC), in the *mce3R* gene. Further analysis of other genes reported to be associated with LZD resistance indicated that non-synonymous mutations were present in all eight oxazolidinone-resistant isolates. Specifically, we identified mutations in the following genes: *tsnR* (Leu232Pro, CTG→CCG), *Rv0545c* (Pro49Ser, CCT→TCT), *Rv0930* (Met5Thr, ATG→ACG, and a premature stop codon at position 305, CGA→TGA), and *Rv3331* (Pro423Leu, CCG→CTG). Additionally, eight drug-resistant isolates harbored non-synonymous mutations of Pro866Ala (CCC→GCC) in *Rv0890c*. Among them, three isolates had a double mutation in Glu234Gly (GAG→GGG), and one had a double mutation in Ser539Asn (AGT→AAT) (Supplementary Table S2).

Although previous studies have reported that mutations in the *rrl*, *rplC*, and *rplD* genes can lead to resistance to oxazolidinones [23,24], our analysis of eight oxazolidinone-resistant isolates revealed that only one isolate, M26, had a non-synonymous mutation in the *rplD* gene. To further investigate the mechanism of oxazolidinone resistance, we performed WGS analysis on the other 169 susceptible isolates. The analysis revealed that 4.73% (8/169 isolates) of the susceptible isolates harbored substitution mutations in the *rrl* gene with G2468T (n = 4), T1152C (n = 2), A2296T (n = 1), and C503T (n = 1). In contrast, 1.78% (3/169 isolates) of sensitive isolates harbored two non-synonymous mutations in the *rplC* gene with Arg44Leu (CGC→CTC, n = 2) and Cys154Arg (TGT→CGT, n = 1). Additionally, 4.14% (7/169 isolates) of sensitive isolates harbored non-synonymous mutations in the *rplD* gene, with six isolates sharing the same mutation as isolate M26, while one isolate had a different non-synonymous mutation, Ser14Arg (AGC→AGA).

## 4. Discussion

The treatment of MDR-TB and XDR-TB remains a global challenge [25]. The value of LZD as a core therapeutic option for MDR-TB has stimulated interest in oxazolidinone antibiotics. However, the severe adverse effects of LZD in clinical use have led to poorer treatment outcomes in patients with M/XDR-TB [26], highlighting the need for safer and more tolerable alternatives. Several oxazolidinones, including TZD, CZD, SZD, and DZD, have shown promising preclinical data. Compared to LZD, TZD has shown better tolerability and safety in both in vivo and in vitro studies [27,28,29]. In a 12-week study, TZD was well tolerated in 44 patients treated for bone and joint infections, with no hematological or neurological adverse effects observed [29]. CZD has similar bactericidal activity to LZD and a favorable safety profile [30,31,32]. In a study of 25 Chinese patients with MDR/RR-TB treated with CZD, 90% of adverse effects associated with LZD were resolved or improved [33]. Several studies have shown that SZD has superior in vitro activity to LZD and is significantly safer [34,35,36,37]. A study by the TB Alliance in healthy volunteers found that SZD was well tolerated at doses ranging from 300 mg to 1800 mg, with no significant adverse effects reported [37]. DZD has demonstrated excellent pharmacokinetics and good safety [38]. A 7-day multiple ascending dose study found that doses ranging from 400 mg to 1600 mg were associated with only mild adverse effects [39]. In a further 21-day study of twice-daily doses of 800 mg or 1200 mg, no serious adverse effects were observed [40]. Given the need for long-term treatment of drug-resistant TB, further studies are urgently needed to assess the efficacy and safety of oxazolidinones in treating these patients.

In this study, we first compared the in vitro activities of these five oxazolidinone drugs (LZD, TZD, CZD, SZD, and DZD) against MDR and pre-XDR-TB isolates. Our data showed that SZD had the strongest antibacterial activity against clinical MTBH isolates, followed by TZD, while CZD and DZD had equivalent and the weakest antibacterial activity (SZD_MIC50_ = TZD_MIC50_ < LZD_MIC50_ < CZD_MIC50_ = DZD_MIC50_; SZD_MIC90_ < TZD_MIC90_ = LZD_MIC90_ < CZD_MIC90_ = DZD_MIC90_). This finding is consistent with a previous study by Wang et al. [41], although another study reported that TZD had the strongest antibacterial activity, with SZD and MRX-I having similar activity to LZD [30]. However, whether such in vitro results reflect in vivo efficacy requires further study. In addition, we proposed the ECOFFs for these drugs as follows: SZD, 0.5 µg/mL; TZD and CZD, 1.0 µg/mL; and DZD, 2.0 µg/mL. Notably, the ECOFF value for DZD was consistent with a previous study by Pang et al. [23], whereas the values for TZD and SZD were 4-fold and 2-fold higher than those proposed by Wang et al. (0.25 µg/mL and 0.125 µg/mL, respectively), respectively, and 2-fold lower (2 µg/mL and 4 µg/mL, respectively) for CZD and DZD [41]. The discrepancies in these results may be due to differences in the types of clinical isolates included and the drug concentrations used to determine the MIC.

The mechanisms of oxazolidinone resistance in clinical MTB isolates are similar to those of LZD, mainly involving ribosomal mutations (*rrl*, *rplC*, *rplD*, and *tsnR*) and non-ribosomal mechanisms such as efflux (*mce3R*, *Rv0545c*, *Rv0930*, *Rv3331,* and *Rv0890c*). However, in this study, no non-synonymous mutations in *rrl* or *rplC* were found in eight oxazolidinone-resistant isolates (Table 3). In contrast, mutation rates of 4.73% and 1.78% were observed in these genes in oxazolidinone-susceptible isolates (Table 4). Aiko et al. [42] also reported that resistant strains showed no known *rrl* or *rplC* mutations associated with LZD resistance, suggesting the presence of alternative resistance mechanisms. Among the eight resistant isolates, two (M26, resistant to TZD and CZD; P604, resistant to all five oxazolidinones) showed distinct mutation patterns in *rplD*, *mce3R*, and *Rv0890c*, which may explain their cross-resistance, needing further experimental validation. Additionally, mutations in the *tsnR*, *Rv0545c*, *Rv0930*, and *Rv3331* genes were identified as potentially linked to LZD resistance (Supplementary Table S2); however, these mutations were also present in susceptible isolates, possibly reflecting lineage-specific variations compared to the reference genome rather than conferring resistance.

We acknowledge several obvious limitations of our study. First, the number of MTB isolates in this experiment was only 177, which may affect the reliability of the ECOFFs defined in this experiment. In addition, the determination of critical concentrations for TZD, CZD, SZD, and DZD should not only be based on ECOFF values but also on pharmacokinetic/pharmacodynamic and clinical outcome data as evaluated in prospective studies [43]. Second, the low rate of oxazolidinone-resistant strains did not allow for an objective assessment of the relationship between the patient’s clinical information (gender, age, occupation, species type, and drug susceptibility) and mutations in known resistance genes and oxazolidinone resistance.

## 5. Conclusions

This study provides the first report on the resistance of MDR-TB isolates in Hainan to oxazolidinones (LZD, TZD, CZD, SZD, and DZD). We found that SZD and TZD exhibited stronger antibacterial activity against MTB than LZD, while CZD and DZD showed comparatively weaker activity. Although in vitro antibiotic efficacy does not always correlate with in vivo results, our findings support the clinical application of SZD and TZD for the treatment of drug-resistant tuberculosis.

## Figures and Tables

**Table 1:** Characteristics of study participants from which the isolate was recovered.

Characteristics	Frequency	Percent (%)
Gender		
Male	142	80.2
Female	35	19.8
Age		
≤18	2	1.1
19–59	141	79.7
≥60	34	19.2
Category		
New	47	26.6
Retreatment	130	73.4
Occupation		
Farmer	110	62.1
Other	67	47.9
Type of specimen		
Sputum	165	93.2
Bronchoalveolar lavage fluid	11	6.2
Joint fluid	1	0.6
Resistance to drugs		
EMB	110	62.1
PTO	25	14.1
CPM	22	12.4
KM	53	29.9

Abbreviations: Other: students, retirees, public officials, etc. EMB: Ethambutol; PTO: Protionamide; CPM: Capreomycin; KM: Kanamycin.

**Table 2:** Distribution of MTB isolates with different LZD, TZD, CZD, SZD, and DZD MIC values.

Classification and Drugs	No. (%) of Isolates with MIC (µg/mL)													
	0.032	0.063	0.125	0.25	0.5	1	2	4	8	16	32	Total	MIC_50_	MIC_90_
	(µg/mL)	(µg/mL)	(µg/mL)	(µg/mL)	(µg/mL)	(µg/mL)	(µg/mL)	(µg/mL)	(µg/mL)	(µg/mL)	(µg/mL)		(µg/mL)	(µg/mL)
MDR														
LZD	0 (0.0)	3 (4.5)	2 (3.0)	33 (49.3)	28 (41.8)	1 (1.5)	0 (0.0)	0 (0.0)	0 (0.0)	0 (0.0)	0 (0.0)	67	0.25	0.5
TZD	4 (6.0)	9 (13.4)	16 (23.9)	28 (41.8)	8 (11.9)	2 (3.0)	0 (0.0)	0 (0.0)	0 (0.0)	0 (0.0)	0 (0.0)	67	0.125	0.5
CZD	0 (0.0)	2 (3.0)	4 (6.0)	13 (19.4)	40 (59.7)	7 (10.4)	1 (1.5)	0 (0.0)	0 (0.0)	0 (0.0)	0 (0.0)	67	0.5	1.0
SZD	3 (4.5)	10 (14.9)	28 (41.8)	21 (31.3)	5 (7.5)	0 (0.0)	0 (0.0)	0 (0.0)	0 (0.0)	0 (0.0)	0 (0.0)	67	0.125	0.25
DZD	0 (0.0)	0 (0.0)	2 (3.0)	7 (10.4)	25 (37.3)	29 (43.3)	4 (6.0)	0 (0.0)	0 (0.0)	0 (0.0)	0 (0.0)	67	0.5	1.0
Pre-XDR														
LZD	1 (0.9)	8 (7.3)	21 (19.1)	46 (41.8)	26 (23.6)	7 (6.4)	1 (0.9)	0 (0.0)	0 (0.0)	0 (0.0)	0 (0.0)	110	0.25	0.5
TZD	11 (10.0)	22 (20.0)	35 (31.8)	23 (20.9)	11 (10.0)	4 (3.6)	3 (2.7)	1 (0.9)	0 (0.0)	0 (0.0)	0 (0.0)	110	0.125	1.0
CZD	0 (0.0)	6 (5.5)	8 (7.3)	48 (43.6)	33 (30.0)	10 (9.1)	2 (1.8)	1 (0.9)	0 (0.0)	1 (0.9)	1 (0.9)	110	0.25	0.5
SZD	16 (14.5)	25 (22.7)	41 (37.3)	23 (20.9)	4 (3.6)	1 (0.9)	0 (0.0)	0 (0.0)	0 (0.0)	0 (0.0)	0 (0.0)	110	0.125	0.25
DZD	0 (0.0)	2 (1.8)	9 (8.2)	23 (20.9)	44 (40.0)	25 (22.7)	6 (5.5)	1 (0.9)	0 (0.0)	0 (0.0)	0 (0.0)	110	0.5	1.0
Total														
LZD	1 (0.6)	11 (6.2)	23 (13.0)	79 (44.6)	54 (30.5)	8 (4.5)	1 (0.6)	0 (0.0)	0 (0.0)	0 (0.0)	0 (0.0)	177	0.25	0.5
TZD	15 (8.5)	31 (17.5)	51 (28.8)	51 (28.8)	19 (10.7)	6 (3.4)	3 (1.7)	1 (0.6)	0 (0.0)	0 (0.0)	0 (0.0)	177	0.125	0.5
CZD	0 (0.0)	8 (4.5)	12 (6.8)	61 (34.5)	73 (41.2)	17 (9.6)	3 (1.7)	1 (0.6)	0 (0.0)	1 (0.6)	1 (0.6)	177	0.5	1.0
SZD	19 (10.7)	35 (19.8)	69 (39.0)	44 (24.9)	9 (5.1)	1 (0.6)	0 (0.0)	0 (0.0)	0 (0.0)	0 (0.0)	0 (0.0)	177	0.125	0.25
DZD	0 (0.0)	2 (1.1)	11 (6.2)	30 (16.9)	69 (39.0)	54 (30.5)	10 (5.6)	1 (0.6)	0 (0.0)	0 (0.0)	0 (0.0)	177	0.5	1

**Table 3:** MICs and *rrl* (23S rRNA), *rplC*, *rplD,* and *mce3R* mutations for the 8 oxazolidinone-resistant clinical isolates.

Isolate	Resistance Genotype				MIC (µg/mL)				
ID	*rrl*	*rplC*	*rplD*	*mce3R*	LZD	TDZ	CZD	SZD	DZD
M26	WT	WT	G236A (R79H)	T217G (Y73D)	0.125	4	4	0.25	1
M108	WT	WT	WT	ins_C −789/−109	1	0.5	2	0.5	2
M143	WT	WT	WT	ins_C −789/−109	1	0.063	32	0.125	1
M161	WT	WT	WT	ins_C −789/−109	1	1	2	0.5	2
M188	WT	WT	WT	ins_C −789/−109	0.5	0.063	16	0.063	0.25
X18	WT	WT	WT	ins_C −789/−109	0.5	2	1	0.125	1
P585	WT	WT	WT	ins_C −789/−109	0.5	2	0.5	0.125	1
P604	WT	WT	WT	ins_C −789/−109	2	2	2	1	4
				C-T −723/−175					

The nucleotide and base positions of the mutations are listed according to MTB H37Rv numbering. Abbreviations: WT, wild type; R, Arg; H, His; Y, Tyr; D, Asp; L, Leu; P, Pro.

**Table 4:** Mutations located in *rrl*, *rplC*, and *rplD* in 18 clinical MTB isolates and MICs of five oxazolidinones.

Isolate	Resistance Genotype			MIC (µg/mL)				
ID	*rrl*	*rplC*	*rplD*	LZD	TDZ	CZD	SZD	DZD
M30	WT	G131T (R44L)	WT	0.063	0.063	0.25	0.125	0.5
M48	WT	T460C (C154R)	WT	0.5	0.125	0.5	0.063	1
M133	WT	WT	C438A (S14R)	0.25	0.125	0.25	0.125	0.5
M140	WT	WT	G236A (R79H)	0.25	0.125	0.5	0.063	0.5
M160	WT	WT	G236A (R79H)	0.032	0.032	0.063	0.032	0.125
M168	WT	WT	G236A (R79H)	0.25	0.25	0.5	0.125	0.5
M199	WT	WT	G236A (R79H)	0.5	0.25	0.5	0.25	1
P507	WT	WT	G236A (R79H)	0.25	0.25	0.25	0.125	0.25
P511	WT	G131T (R44L)	WT	0.25	0.063	0.5	0.125	0.25
P520	WT	WT	G236A (R79H)	0.25	0.063	0.25	0.125	0.25
P536	G2468T	WT	WT	0.25	0.125	0.5	0.125	0.25
P561	G2468T	WT	WT	0.25	0.5	0.5	0.25	0.25
P562	G2468T	WT	WT	0.25	0.5	0.5	0.125	0.25
P566	G2468T	WT	WT	0.25	0.125	0.5	0.125	0.25
P568	T1152C	WT	WT	0.063	0.032	0.063	0.032	0.063
P570	T1152C	WT	WT	0.5	0.5	0.5	0.25	0.5
P579	A2296T	WT	WT	0.5	0.032	0.125	0.063	0.5
P603	C503T	WT	WT	0.125	0.063	0.25	0.063	0.125

The nucleotide and base positions of the mutations are listed according to MTB H37Rv numbering. Abbreviations: WT, wild type; R, Arg; L, Leu; C, Cys; S, Ser; H, His.

## Data Availability

No datasets were generated or analyzed during the current study. The original contributions presented in this study are included in the article/Supplementary Material; further inquiries can be directed to the corresponding authors.

## Supplementary Materials

The following supporting information can be downloaded at https://www.mdpi.com/article/10.3390/pathogens14030218/s1, Table S1: The basic information for the patients with 177 isolates. Table S2: MICs and *tsnR*, *Rv0545c*, *Rv0890c*, *Rv0930,* and *Rv3331* mutations for the 8 oxazolidinone-resistant clinical isolates.
