# Supplementary material for: Comparative In Vitro Drug Susceptibility Study of Five Oxazolidinones Against Mycobacterium tuberculosis in Hainan, China
Source: Pathogens. 2025 Feb 24;14(3):218. doi: 10.3390/pathogens14030218 (PMC11945096; doi:10.3390/pathogens14030218)
Supplement: Supplementary file 1 [file pathogens-14-00218-s001.zip › pathogens-3464353-supplementary.pdf]

Table S1. The basic information for the patients with 177 isolates.

Isolate ID	Sex	Age	Category	Type of specimen	Occupation	EMB	PTO	CPM	KM
M1	M	67	New	SP	Farmer	S	S	S	S
M2	M	62	Re	SP	Farmer	R	S	R	R
M3	M	59	Re	SP	Farmer	S	S	S	S
M4	M	61	N	SP	Farmer	S	S	S	S
M5	F	44	N	SP	Farmer	S	S	S	S
M6	F	50	Re	SP	Farmer	R	S	S	S
M7	M	51	Re	SP	Farmer	S	R	R	R
M8	M	65	N	SP	Farmer	S	R	S	S
M9	M	47	N	SP	Farmer	S	R	S	S
M11	M	70	Re	SP	Farmer	R	S	S	S
M12	M	45	N	SP	Farmer	S	S	S	S
M14	M	61	Re	SP	Farmer	S	S	S	S
M15	M	62	Re	SP	Farmer	R	S	S	S
M16	M	53	Re	SP	Other	S	R	S	S
M18	M	30	Re	JF	Farmer	R	S	S	S
M19	M	37	N	SP	Farmer	R	R	S	S
M20	M	60	Re	SP	Other	S	S	S	S
M21	M	67	Re	SP	Other	S	S	S	S
M22	M	59	Re	SP	Farmer	R	S	S	S
M23	M	49	Re	SP	Farmer	R	S	S	S
M24	M	65	Re	SP	Other	R	S	S	S

Table S1. Cont.

Isolate ID	Sex	Age	Category	Type of specimen	Occupation	EMB	PTO	CPM	KM
M25	M	65	Re	SP	Farmer	S	S	S	S
M26	M	77	N	SP	Other	S	S	S	S
M28	M	38	N	SP	Farmer	S	S	S	S
M30	M	46	Re	SP	Other	R	R	S	S
M31	F	65	N	SP	Other	S	S	S	S
M32	M	28	Re	SP	Farmer	S	S	S	S
M34	M	47	Re	SP	Farmer	R	S	S	S
M35	M	64	Re	SP	Other	S	S	S	S
M37	F	55	Re	SP	Farmer	S	S	S	S
M38	M	61	Re	SP	Farmer	R	R	R	R
M39	M	19	Re	SP	Farmer	R	S	S	S
M40	M	48	N	SP	Farmer	S	S	S	S
M41	M	52	Re	SP	Farmer	R	S	S	S
M42	M	68	Re	SP	Farmer	S	S	S	S
M43	M	22	Re	SP	Farmer	S	S	S	S
M44	M	54	N	SP	Other	S	S	S	S
M45	M	66	Re	SP	Farmer	S	S	S	S
M46	M	40	Re	SP	Farmer	S	S	S	S
M47	M	48	Re	SP	Farmer	R	R	S	S
M48	M	66	Re	SP	Farmer	S	S	S	S
M49	M	53	N	SP	Farmer	S	S	S	S

Table S1. Cont.

Isolate ID	Sex	Age	Category	Type of specimen	Occupation	EMB	PTO	CPM	KM
M50	M	46	Re	SP	Farmer	S	S	S	S
M51	M	43	Re	SP	Farmer	S	S	S	S
M52	M	58	Re	SP	Farmer	R	S	S	S
M54	M	34	Re	SP	Farmer	S	S	S	S
M55	M	65	Re	SP	Farmer	R	S	R	R
M57	M	60	N	SP	Farmer	R	S	S	S
M59	M	36	Re	SP	Other	R	S	S	S
M60	M	45	Re	SP	Other	S	S	S	S
M61	M	64	Re	SP	Farmer	S	S	S	S
M62	M	36	Re	SP	Farmer	S	R	S	S
M63	M	42	Re	SP	Farmer	S	S	S	S
M64	M	57	Re	SP	Other	S	R	R	S
M66	M	64	Re	SP	Other	R	R	S	S
M67	M	52	N	SP	Farmer	S	S	S	S
M68	F	23	Re	BALF	Farmer	R	S	S	S
M69	M	46	Re	SP	Farmer	S	S	S	S
M70	M	55	Re	SP	Farmer	R	S	S	S
M72	M	75	Re	SP	Farmer	S	S	S	R
M73	F	34	Re	SP	Other	S	S	S	S
M74	M	49	Re	SP	Farmer	S	S	S	S
M75	M	48	Re	SP	Farmer	S	S	S	S

Table S1. Cont.

Isolate ID	Sex	Age	Category	Type of specimen	Occupation	EMB	PTO	CPM	KM
M77	M	42	N	SP	Farmer	R	S	S	S
M78	M	70	Re	SP	Farmer	R	R	S	S
M79	M	54	Re	SP	Farmer	R	S	S	S
M80	M	52	Re	SP	Farmer	R	R	R	R
M81	M	68	Re	SP	Farmer	S	S	R	S
M82	M	56	Re	SP	Other	S	S	S	S
M84	M	45	Re	SP	Farmer	S	S	S	S
M85	M	58	Re	SP	Farmer	R	S	S	S
M86	M	47	Re	SP	Farmer	S	S	S	S
M88	F	46	Re	SP	Farmer	R	R	S	S
M89	M	23	Re	SP	Farmer	R	S	S	S
M92	M	36	Re	SP	Farmer	S	S	S	S
M94	M	59	Re	SP	Other	R	R	S	R
M102	M	48	N	SP	Farmer	S	S	S	S
M105	M	47	Re	SP	Farmer	R	S	S	S
M107	F	32	Re	SP	Farmer	R	S	S	S
M108	M	54	N	SP	Farmer	S	S	S	S
M109	M	50	Re	SP	Farmer	R	S	S	S
M110	M	36	Re	SP	Other	R	S	S	S
M112	M	48	N	SP	Farmer	S	S	S	S
M113	F	54	Re	SP	Other	R	S	S	S

Table S1. Cont.

Isolate ID	Sex	Age	Category	Type of specimen	Occupation	EMB	PTO	CPM	KM
M120	F	21	N	SP	Other	R	S	S	S
M121	M	44	Re	SP	Other	S	S	S	S
M122	M	60	Re	SP	Farmer	R	S	R	R
M126	F	37	Re	SP	Farmer	R	R	R	R
M128	F	25	Re	SP	Other	R	S	S	S
M133	F	76	Re	SP	Farmer	S	S	S	S
M134	F	19	N	SP	Other	R	S	S	S
M140	M	76	Re	SP	Farmer	R	S	S	S
M143	F	28	Re	SP	Farmer	R	R	S	S
M148	M	22	N	SP	Other	R	S	S	S
M150	M	30	Re	SP	Farmer	R	S	S	S
M155	M	59	Re	SP	Farmer	S	S	S	S
M158	M	56	Re	SP	Farmer	R	S	S	S
M160	M	29	Re	SP	Other	R	R	R	R
M161	M	59	N	SP	Other	S	S	S	S
M168	M	59	Re	SP	Farmer	S	S	S	S
M169	M	58	Re	SP	Farmer	R	R	S	S
M175	M	58	Re	SP	Farmer	R	S	S	S
M178	M	54	Re	SP	Farmer	R	S	S	S
M180	M	59	Re	SP	Other	R	S	R	R
M185	M	49	N	SP	Farmer	S	S	S	S

Table S1. Cont.

Isolate ID	Sex	Age	Category	Type of specimen	Occupation	EMB	PTO	CPM	KM
M187	M	58	Re	SP	Farmer	R	S	S	S
M188	M	80	Re	SP	Farmer	R	S	S	S
M196	M	39	Re	SP	Farmer	R	S	S	S
M197	M	68	Re	SP	Other	R	S	S	S
M198	F	19	Re	SP	Farmer	R	S	R	R
M199	M	52	Re	SP	Farmer	S	S	S	S
M201	M	42	Re	SP	Farmer	S	S	S	S
M205	M	43	Re	SP	Farmer	R	S	S	S
M206	M	30	N	SP	Farmer	S	S	S	S
M207	F	54	N	SP	Other	R	R	S	S
M212	M	20	N	SP	Farmer	R	S	R	R
M213	M	50	Re	SP	Farmer	R	S	R	R
M215	F	48	Re	BALF	Other	S	R	R	S
M216	M	32	N	BALF	Farmer	R	S	S	S
M222	M	42	Re	SP	Farmer	S	S	S	S
M224	M	31	Re	SP	Other	R	S	S	S
X11	F	34	Re	SP	Farmer	R	S	R	R
X12	M	47	N	SP	Farmer	R	S	R	R
X13	F	23	Re	SP	Other	S	S	S	R
X17	M	53	Re	SP	Farmer	R	S	R	S
X18	F	41	Re	SP	Farmer	S	S	R	S

Table S1. Cont.

Isolate ID	Sex	Age	Category	Type of specimen	Occupation	EMB	PTO	CPM	KM
P507	M	41	Re	SP	Farmer	R	S	S	R
P508	F	65	N	SP	Farmer	R	R	S	R
P510	F	25	Re	SP	Other	R	S	S	R
P511	M	49	Re	SP	Farmer	R	S	S	R
P518	M	30	Re	SP	Other	R	S	S	R
P519	F	35	N	SP	Other	R	S	S	R
P520	M	58	Re	SP	Farmer	R	S	S	R
P521	M	48	N	SP	Worker	R	S	S	R
P523	M	55	Re	SP	Farmer	R	S	S	R
P526	M	74	Re	SP	Other	R	S	S	R
P532	F	42	N	SP	Other	R	S	R	R
P536	M	61	Re	SP	Farmer	R	S	S	R
P537	M	56	Re	SP	Farmer	R	S	S	R
P538	M	48	Re	SP	Other	R	S	S	R
P539	M	51	Re	SP	Other	R	S	S	R
P543	F	58	Re	SP	Other	R	S	R	R
P544	F	31	N	SP	Other	R	S	S	R
P545	M	73	N	SP	Other	R	S	S	R
P546	M	52	Re	BALF	Other	R	S	S	R
P549	F	63	Re	BALF	Other	R	S	S	R
P550	M	46	N	SP	Other	R	R	S	R

Table S1. Cont.

Isolate ID	Sex	Age	Category	Type of specimen	Occupation	EMB	PTO	CPM	KM
P551	M	29	Re	SP	Other	R	S	S	R
P552	M	71	N	SP	Farmer	R	S	S	R
P556	M	35	Re	SP	Other	R	R	S	R
P560	M	53	Re	BALF	Other	R	S	S	R
P561	F	69	N	SP	Other	R	S	S	R
P562	M	47	N	SP	Farmer	R	S	S	R
P566	M	34	Re	SP	Other	R	S	S	R
P567	M	24	N	SP	Other	R	S	S	R
P568	M	47	Re	SP	Other	R	S	S	R
P570	M	68	N	BALF	Farmer	R	S	S	R
P572	M	45	Re	SP	Other	R	S	S	R
P576	M	53	Re	SP	Other	R	S	S	R
P577	F	15	Re	SP	Other	R	S	S	R
P579	M	57	Re	SP	Farmer	R	S	S	R
P580	F	49	Re	SP	Other	R	S	R	R
P582	M	61	Re	BALF	Other	R	S	S	S
P583	M	55	N	SP	Farmer	S	S	S	S
P584	F	26	N	SP	Other	S	S	S	S
P585	M	53	Re	SP	Other	R	S	S	S
P587	F	35	N	SP	Other	R	S	S	S
P590	M	16	N	SP	Other	R	S	S	S

Table S1. Cont.

Isolate ID	Sex	Age	Category	Type of specimen	Occupation	EMB	PTO	CPM	KM
P591	F	70	Re	SP	Farmer	S	S	S	S
P593	M	51	N	BALF	Farmer	R	S	S	S
P594	M	29	Re	SP	Other	R	S	S	S
P597	M	55	Re	SP	Other	R	R	S	S
P598	M	29	N	BALF	Other	S	S	S	S
P599	F	35	Re	SP	Other	S	S	S	S
P600	M	68	Re	BALF	Farmer	R	S	S	S
P603	M	54	Re	SP	Other	R	S	S	S
P604	M	77	Re	SP	Other	R	S	S	S

Abbreviations: F, Female; M, Male; N, New treatment; Re, Re-treatment; SP, Sputum; BALF, Bronchoalveolar lavage fluid; JF, Joint fluid; Other: Students, retirees, public officials, etc. R, Resistant; S, Susceptible; EMB: Ethambutol; PTO: Protionamide; CPM: Capreomycin; KM: Kanamycin.

Table S2. MICs and *tsnR*, *Rv0545c*, *Rv0890c*, *Rv0930* and *Rv3331* mutations for the 8 oxazolidinone-resistant clinical isolates

**Isolate**	**Resistance genotype**					**MIC (μg/mL)**				
**ID**	***tsnR***	***Rv0545c***	***Rv0890c***	***Rv0930***	***Rv3331***	**LZD**	**TDZ**	**CZD**	**SZD**	**DZD**
M26	T695C (L232P)	C145T (P49S)	C2596G (P866A)<br>G1616A (S539N)	T14C (M5T)<br>C913T (305R-stop)	C1268T (P423L)	0.125	4	4	0.25	1
M108	T695C (L232P)	C145T (P49S)	C2596G (P866A)	T14C (M5T)<br>C913T (305R-stop)	C1268T (P423L)	1	0.5	2	0.5	2
M143	T695C (L232P)	C145T (P49S)	C2596G (P866A)<br>A701G (E234G)	T14C (M5T)<br>C913T (305R-stop)	C1268T (P423L)	1	0.063	32	0.125	1
M161	T695C (L232P)	C145T (P49S)	C2596G (P866A)<br>A701G (E234G)	T14C (M5T)<br>C913T (305R-stop)	C1268T (P423L)	1	1	2	0.5	2
M188	T695C (L232P)	C145T (P49S)	C2596G (P866A)	T14C (M5T)<br>C913T (305R-stop)	C1268T (P423L)	0.5	0.063	16	0.063	0.25
X18	T695C (L232P)	C145T (P49S)	C2596G (P866A)	T14C (M5T)<br>C913T (305R-stop)	C1268T (P423L)	0.5	2	1	0.125	1
P585	T695C (L232P)	C145T (P49S)	C2596G (P866A)<br>A701G (E234G)	T14C (M5T)<br>C913T (305R-stop)	C1268T (P423L)	0.5	2	0.5	0.125	1
P604	T695C (L232P)	C145T (P49S)	C2596G (P866A)	T14C (M5T)<br>C913T (305R-stop)	C1268T (P423L)	2	2	2	1	4

The nucleotide and base positions of the mutations are listed according to MTB H37Rv numbering.

Abbreviations: L, Leu; P, Pro; S, Ser; N, Asn; A, Ala; E, Glu; G, Gly; M, Met; T, Thr; R, Arg;
